# Bayesian population structure analysis reveals presence of phylogeographically specific sublineages within previously ill-defined T group of *Mycobacterium tuberculosis*

**DOI:** 10.1371/journal.pone.0171584

**Published:** 2017-02-06

**Authors:** Yann Reynaud, Chao Zheng, Guihui Wu, Qun Sun, Nalin Rastogi

**Affiliations:** 1 WHO Supranational TB Reference Laboratory, Tuberculosis and Mycobacteria Unit, Institut Pasteur de la Guadeloupe, Morne Jolivière Abymes, Guadeloupe, France; 2 College of Life Sciences, Sichuan University, Chengdu, Sichuan, China; 3 Chengdu Public Health Clinical Center, Chengdu, Sichuan, China; University of Minnesota, UNITED STATES

## Abstract

*Mycobacterium tuberculosis* genetic structure, and evolutionary history have been studied for years by several genotyping approaches, but delineation of a few sublineages remains controversial and needs better characterization. This is particularly the case of T group within lineage 4 (L4) which was first described using spoligotyping to pool together a number of strains with ill-defined signatures. Although T strains were not traditionally considered as a real phylogenetic group, they did contain a few phylogenetically meaningful sublineages as shown using SNPs. We therefore decided to investigate if this observation could be corroborated using other robust genetic markers. We consequently made a first assessment of genetic structure using 24-loci MIRU-VNTRs data extracted from the SITVIT2 database (n = 607 clinical isolates collected in Russia, Albania, Turkey, Iraq, Brazil and China). Combining Minimum Spanning Trees and Bayesian population structure analyses (using STRUCTURE and TESS softwares), we distinctly identified eight tentative phylogenetic groups (T1-T8) with a remarkable correlation with geographical origin. We further compared the present structure observed with other L4 sublineages (n = 416 clinical isolates belonging to LAM, Haarlem, X, S sublineages), and showed that 5 out of 8 T groups seemed phylogeographically well-defined as opposed to the remaining 3 groups that partially mixed with other L4 isolates. These results provide with novel evidence about phylogeographically specificity of a proportion of ill-defined T group of *M*. *tuberculosis*. The genetic structure observed will now be further validated on an enlarged worldwide dataset using Whole Genome Sequencing (WGS).

## Introduction

*Mycobacterium tuberculosis* genetic structure, dispersal and evolution have been explored for years by genotyping [1]. Several well-known approaches are today available such as IS*6110*-RFLP [2], CRISPRs (Clustered Regularly Interspaced Short Palindromic Repeats)–based spoligotyping [3], MIRU-VNTRs (Mycobacterial Interspersed Repetitive Unit—Variable Number of Tandem Repeats) [4], and RD-LSPs (Regions of Differences–Large Sequence Polymorphisms) [5]. The last approach was used to classify *M*. *tuberculosis* complex into six major lineages: Lineage 1 (Indo-Oceanic), Lineage 2 (East-Asian including Beijing), Lineage 3 (East-African-Indian), Lineage 4 (Euro-American), Lineage 5 (West Africa or *M*. *africanum* I), and Lineage 6 (West Africa or *M*. *africanum* II). Another Lineage 7 was since described in Ethiopia and the Horn of Africa [6]. Lastly, a robust SNP barcode (Single Nucleotide Polymorphism) was also developed based on WGS [1]. Depending on the purpose of a genotyping, all these approaches have advantages and inconveniences. For example SNPs calling has the highest discriminatory power to explore sublineages; nevertheless waiting for real democratization of this tool, it is still not used for epidemiological surveys in most of the countries. On the opposite, despite reported discrepancies in *M*. *tuberculosis* structuring due to inherent homoplasy (occurring through convergence, reverse evolution, and horizontal gene transfer) and low mutation rates [1,7,8] of the genetic loci analyzed by spoligotyping, this method is still widely used in association with MIRU-VNTRs for global epidemiological surveys.

In the above context, classification of certain sublineages, particularly the T group within lineage 4 (L4, which also comprises LAM, H, X and S sublineages), is yet poorly understood and still subject to debate. Based on spoligotyping, the so-called term “T lineage” was initially coined to pool together a number of ill-defined spoligotyping signatures such as T1 to T5 [9] and T-Tuscany [10], and later expanded to include other sublineages even though some were better defined phylogeographically as reviewed in SITVITWEB [11]; examples include T1-RUS2 and T5-RUS1 (Russia), T2-Uganda, T3-ETH (Ethiopia) [12], T3-OSA (Japan) [13], T4-CEU1 (Central Europe) and T5-Madrid2 (Spain) [14]. To summarize, albeit T group includes mostly strains that do not structure together as a phylogenetic group *stricto sensu*, recent studies based on robust SNP markers revealed that they did contain eight phylogenetically meaningful sublineages without mixing with other L4 subgroups, and were numbered 4.4.1.2, 4.4.2, 4.6.1.1, 4.6.1.2, 4.6.2.1, 4.7, 4.8 and 4.9 [1]. Thus even though the initial T group structuring based on spoligotyping alone was considered misleading, it nonetheless paved the way to decipher recent subdivision of T isolates into several potential clusters. We therefore consider that digging into phylogeographical specificity of well-structured T group isolates makes sense. Based on a large international dataset of 24-loci MIRU-VNTR markers (which are less subjected to homoplasy and present higher mutation rate as compared to spoligotyping), we hereby provide novel evidence regarding genetic structure of T group isolates, with a clear-cut phylogeographical specificities for 5 out of 8 sublineages.

## Materials and methods

### Data collection

Anonymized data on *M*. *tuberculosis* T lineage strains genotyped using spoligotyping and 24-loci MIRU-VNTRs were extracted from the SITVIT2 proprietary database of Institut Pasteur de la Guadeloupe [15], which is an updated version of the SITVITWEB database [11]. Most of data were published earlier within a context focusing on *M*. *tuberculosis* population structure and/or epidemiology within a country or region [16–24]. However, for data submitted to the database but not yet published by respective investigators, permission was officially sought and duly granted by following researchers: Dr. Ling Cheng (Department of Respiratory Medicine, Affiliated Hospital of Zunyi Medical College, Zunyi, Guizhou, China), Dr. Silva Tafaj (Microbiology Department, University Hospital "Shefqet Ndroqi", Tirana, Albania), and Dr Nurhan Albayrak / Dr Rýza Durmaz (Department of Microbiology Reference Laboratories, Ministry of Health, Public Health Agency of Turkey, Ankara, Turkey). The T lineage strains studied (n = 607 isolates) were collected from Russia (n = 17), Albania (n = 100), Turkey (n = 72), Iraq (n = 76), Brazil (n = 90) and China (n = 252). Within China, dataset was divided into regions in Tibet (n = 13), Sichuan (n = 83), Guizhou (n = 43), Chongqing (n = 74) and Jiangsu (n = 39).

### Phylogenetic inferences

BioNumerics software 6.6 (Applied Maths, Sint-Martens-Latem, Belgium) was used to visualize evolutionary relationships between the T clinical isolates by drawing Minimum Spanning Trees (MSTs) using 24-loci MIRU-VNTR and spoligotype data. MSTs are undirected graphs in which all samples are connected together with the fewest possible connections between nearest neighbors.

### Bayesian population structure analyses

To explore genetic structure of *M*. *tuberculosis* T isolates, two Bayesian clustering algorithms were used in parallel, implemented in the software STRUCTURE 2.3 [25] and in TESS 2.3 [26, 27]. In both programs, an admixture model was implemented considering that the data originate from the admixture of *k* ancestral populations at some time in the past. The ancestry coefficient (or admixture proportion) in the individual Q-matrix correspond to part of the genome that each individual inherited from ancestors. Admixture models are a common feature for real data and are therefore more flexible than models without admixture. Posterior estimates for the parameters of interest are computed by using a Markov chain Monte Carlo (MCMC) algorithm. In our study, STRUCTURE was run in 10 parallel MCMC for K populations ranging from 3 to 10, with a burn-in of 100000 iterations and a run length of 10^6^ iterations following the burn-in. To estimate the right number of population among T isolates, ln P(D|K) (the logarithm of the probability of the data given K) was calculated using the program STRUCTURE HARVESTER [28], as well as the delta K calculated by the Evanno method [29]. Concerning the TESS analysis, random spatial coordinates were first generated individually (within each country or region) prior to any run. TESS was then run in 10 replicates, with K_max_ ranging from 2 to 15 for 50000 sweeps with a burn-in period of 10000. To estimate the right number of clusters among T dataset, the deviance information criterion (DIC) was computed and plotted against K_max_ [30].

For both STRUCTURE and TESS Q-matrix, medians were then calculated from 10 replicates for K = 8 by using the Greedy algorithm implemented in CLUMPP 1.1.2 software [31] to guarantee the optimum clustering for each analyses. Results of admixture coefficients were then displayed spatially by an interpolation technique called universal kriging: Q-matrix were represented either on a single map (ETOPO1 map produced by NOAA freely available as indicated here: https://www.ngdc.noaa.gov/mgg/global/dem_faq.html#sec-2.4 [32]) using the script 'POPSutilities.r' implemented in the program R, or on separate maps for each K by using the script 'plot.admixture.r' (both scripts available through TESS website: http://membres-timc.imag.fr/Olivier.Francois/tess.html).

New MST analyses were then performed using BioNumerics software 6.6 and identifying *M*. *tuberculosis* T strains belonging to sublineages 1 to 8 defined by STRUCTURE analysis using a cutoff of 0.5. For each sublineage, BlockLogo was used to visualize main patterns of tandem repeats of 24 loci MIRU-VNTRs [33]. The Hunter-Gaston discriminatory index (HGDI) was calculated as previously described [34].

### Allelic richness

For analyses on allelic richness, 24-loci MIRU-VNTR data were grouped according to *M*. *tuberculosis* T sublineages defined by STRUCTURE software. Mean allelic richness was evaluated using statistical technique of rarefaction implemented in the software HP-RARE 1.0 which compensates for sampling disparity [35]. Results were compared based on Dunn’s test for stochastic dominance [36] followed by multiple pairwise comparisons of the stochastic dominance among k groups using the Kruskal-Wallis test [37].

### Ethics statements

None required since the genotyping data were already published or extracted as anonymized data from the SITVIT2 database.

## Results and discussion

### Phylogenetic inference of T lineage isolates by MST

**Fig 1:**
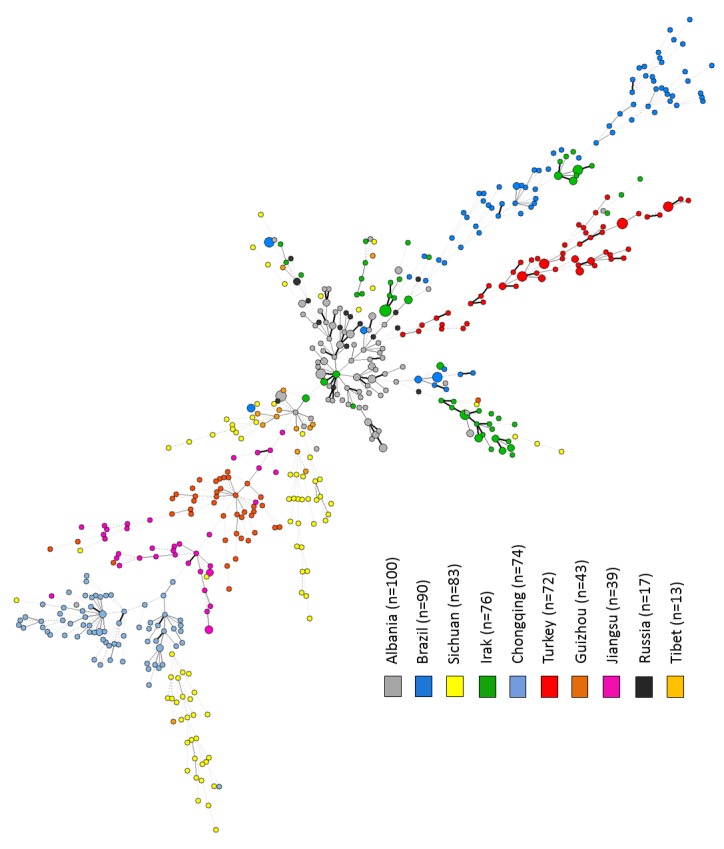
Minimum Spanning Tree (MST) illustrating evolutionary relationships between *M*. *tuberculosis* T lineage isolates (n = 607) based on 24-loci MIRU-VNTR. The MST connects each genotype based on degree of changes required to go from one allele to another; the complexity of the lines denotes the number of allele/spacer changes between two patterns: solid lines (1 or 2 or 3 changes), gray dashed lines (4 changes) and gray dotted lines (5 or more changes); the size of the circle is proportional to the total number of isolates sharing same pattern.

Evolutionary relationships between *M*. *tuberculosis* T isolates were explored by MST analysis. Spoligotyping showed a non-perfect structuring of T sublineages with main central node made up of the T1 (n = 450), T2 (n = 73) and T3 (n = 40) sublineages and other sublineages mixed up in the MST without any clear structure. Furthermore, no clear-cut geographical segregation of T isolates could be highlighted from this spoligotyping data (S1 Fig), corroborating the fact that T sublineages include many strains that do not structure together as a single phylogenetic group *stricto sensu* [11]. Indeed, spoligotyping used alone may misclassify certain strains mainly due to homoplasy and weak mutation rates [1,7,8]. Hence, we further explored evolutionary relationships by constructing a MST focusing on more robust 24-loci MIRU-VNTR of the studied T strains (n = 607 isolates, Fig 1). We observed a surprisingly clear-cut correlation between geographical regions, strains, and phylogenetic groups. Briefly, a visual segregation based on countries was palpable as well as regional differences within a large country like China, although exceptions included existence of two groups in Sichuan, and the observation that some isolates from Iraq clustered with Brazilian strains (Fig 1).

### Bayesian population and spatial analyses

**Fig 2:**
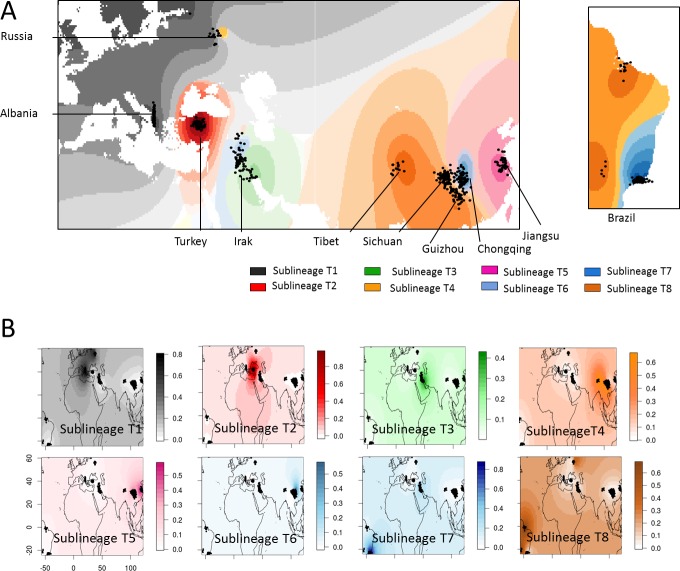
STRUCTURE Ancestry coefficient (Q-matrix) of *M*. *tuberculosis* T isolates displayed spatially by universal kriging. Q-matrix are represented on (A) a single map, or (B) separate maps for each K with density of colors increasing with ancestry coefficient; black dots represent spatial coordinates of individuals.

To better characterize and delineate clusters revealed by 24-loci MIRU-VNTR analysis, we further performed two Bayesian clustering approaches implemented in STRUCTURE 2.3 [25] and TESS 2.3 softwares [26,27]. STRUCTURE, which explores clusters and clines by making use of multilocus genotypes as for example MIRU-VNTR, is the most influential program since Bayesian revolution [38]. This approach has been improved in several Bayesian spatial clustering programs (as TESS) by adding individual geographic coordinates as prior parameters. The appropriate K value was selected for the STRUCTURE analyses by the lnP(D|K) and the derived delta K calculated by the Evanno method [29] (S2A and S2B Fig) and for the TESS analyses by plotting DIC values against K_max_ (S2C Fig). Congruent results were obtained between both approaches with a total of K = 8 divergent populations named sublineages T1 to T8 (Fig 2 and S3 Fig). Classification of T isolates to each population were very similar between STRUCTURE and TESS approaches except for sublineage T8 for which higher probabilities were obtained in the STRUCTURE analysis, then allowing a better structuring of a few strains in this sublineage (detailed TESS results are available as S4 Fig and S1 Table).

Bayesian population and spatial analyses confirmed surprisingly contrasted geographical distribution of each sublineage (Fig 2 and Table 1): sublineage T1 was predominant in Albania and Russia, representing respectively 77% and 64.7% of total T isolates; T2 represented 100% of T isolates from Turkey; T3 represented 34.2% of T strains in Iraq and 20.5% of T strains in Jiangsu, China; T4 represented 76.9% of T isolates in Tibet, 74.4% in Guizhou and 50.6% in Sichuan; T5 represented 59% of T isolates in Jiangsu, 37.3% in Sichuan and 35.1 in Chongqing; T6 represented 59.5% of T isolates in Chongqing; T7 represented 77.8% of T isolates in Brazil and 34.2% in Iraq; and finally T8 represented 23.5% of T isolates in Russia and 18.9% in Brazil. It is difficult to hypothesize whether such a contrasted phylogeographical patterns of T sublineages evolved due to intricate host-pathogen interactions, or due to respective immigration history of these subpopulations, or both.

**Table 1:** Number of each *M*. *tuberculosis* T sublineages (defined by STRUCTURE analysis) per country or regions in China.

Country & regions	T1 (n = 99)	T2 (n = 73)	T3 (n = 43)	T4 (n = 105)	T5 (n = 84)	T6 (n = 49)	T7 (n = 98)	T8 (n = 28)	Int (n = 28)
ALB (n = 100)	77		3	9		1	2	3	5
Brazil (n = 90)	2						70	17	1
Iraq (n = 76)	9	1	26	5			26	3	6
Turkey (n = 72)		72				0			
Russia (n = 17)	11			1				4	1
Sichuan (n = 83)			3	42	31	1			6
Chongqing (n = 74)				1	26	44			3
Guizhou (n = 43)			3	32	3	2			3
Jiangsu (39)			8	5	23	1			2
Tibet (n = 13)				10	1			1	1

Strains in intermediate position between sublineages are indicated Int.

**Fig 3:**
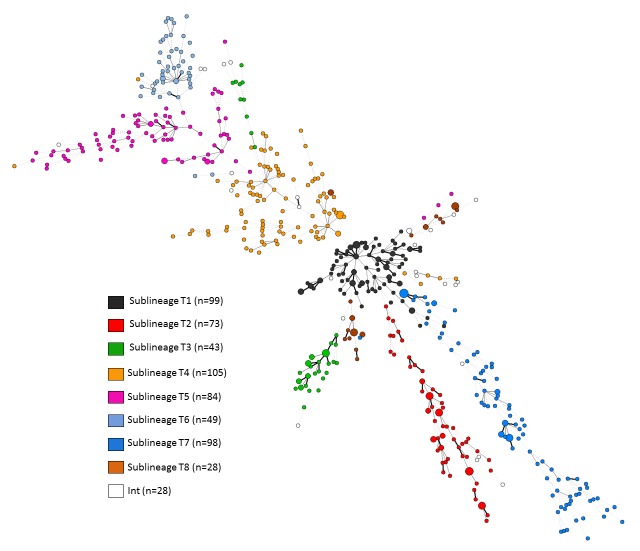
MST based on 24-loci MIRU-VNTR illustrating evolutionary relationships of the T sublineages isolates (n = 607) prelabeled as T1 to T8 based on previous STRUCTURE analysis. Strains in intermediate position between sublineages are indicated as Int. The complexity of the lines denotes the number of allele/spacer changes between two patterns while the size of the circle is proportional to the total number of isolates sharing same pattern.

Nonetheless, this structure was further confirmed by performing a new MST analysis of isolates labeled as sublineages T1 to T8 (Fig 3). Results were very congruent between both approaches except for few isolates belonging to sublineages T8 and T3 in the STRUCTURE analysis, but appearing separated in the MST analysis. These imperfectly structured isolates in T8 and T3 sublineages corresponded to lowest ancestry coefficient in the TESS analysis and classified as being in intermediate position (S3 and S4 Figs, S1 Table), and should be further explored based on WGS.

**Fig 4:**
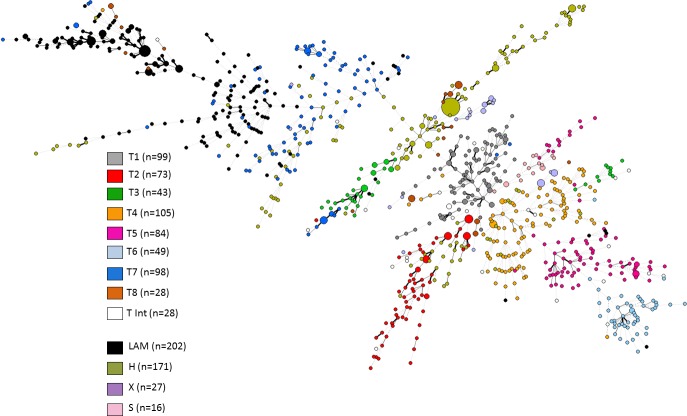
MST based on 24-loci MIRU-VNTR combined with spoligotyping and illustrating evolutionary relationships of the T sublineages isolates (n = 607) prelabeled as T1 to T8 based on previous STRUCTURE analysis, and LAM, H, X and S isolates from SITVIT2 database.

Since T group strains are considered part of the larger Euro-American L4 (which also comprises numerous LAM, H, X and S sublineages), we performed further MST analyses using 24-loci MIRU-VNTRs combined with spoligotyping data in order to perceive evolutionary relationships of an international collection of T group strains (n = 607) prelabeled as T1 to T8 based on STRUCTURE analysis, and LAM, H, X and S isolates (n = 416) from SITVIT2 database (Fig 4). Using this approach, we intended to confirm if the sublineages T1 to T8 really constituted independent groups when compared to other L4 strains. The resulting MST (Fig 4) globally showed that with the exception of T8 isolates which seems to split into several sublineages, and T2 and T7 which clustered with few H and LAM isolates–revealing probable misclassification of these T strains due to homoplasy or artefacts; all other sublineages described were pretty well-structured. We further compared 24-loci MIRU-VNTR profiles of this T sublineages with profiles available in the MIRU-VNTRplus database [39,40], confirming that these T1 to T8 were not related to other L4 isolates available in the database (S5 Fig).

### Genetic characteristics of T sublineages

**Fig 5:**
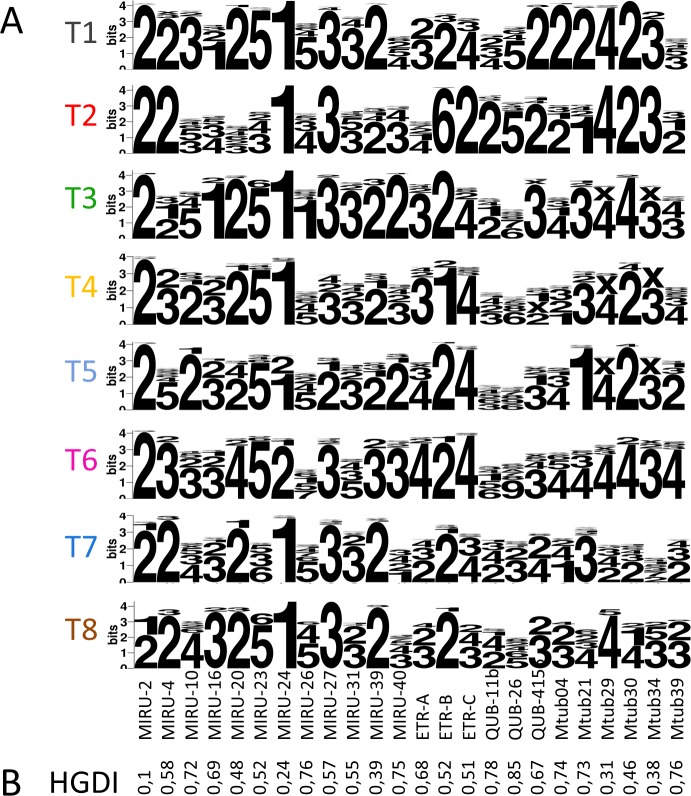
Allele copy numbers and discriminatory index of 24-loci MIRU-VNTR markers. (A) Logo of allele copy number of 24-loci MIRU-VNTR markers in *M*. *tuberculosis* sublineages T1 to T8. X: not done. (B) Hunter-Gaston discriminatory index (HGDI) for each 24-loci MIRU-VNTR markers.

**Fig 6:**
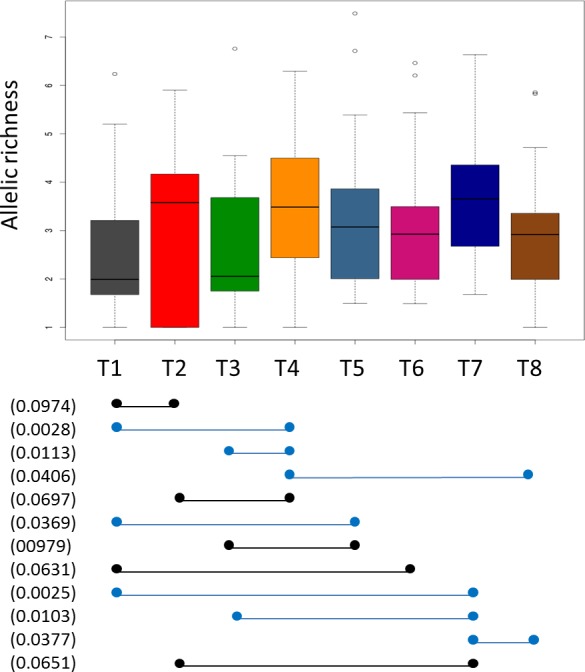
Boxplot of allelic richness of *M*. *tuberculosis* T sublineages T1 to T8 calculated by a rarefaction procedure implemented in HP-RARE 1.0 software. Significant differences calculated by the Dunn’s test at p-values<0.05 are indicated by blue line, and p-value<0.1 by black line. P-value in parenthesis. Boxes correspond to median values ± quartiles of allelic richness; adjacent lines show the minimum/maximum values; dots represent outlier values.

When focusing on markers driving structuring of T sublineages, one can define the predominant tandem repeat numbers encountered in each sublineage (Fig 5 and S3 Table). 24-loci MIRU-VNTR mean allelic richness was calculated by a rarefaction procedure correcting for sample size effects and implemented in the software HP-RARE 1.0 [35] (Fig 6). Some differences between sublineages were highlighted by Dunn’s test (Fig 6 and S2 Table): sublineages T4 and T7 presented higher allelic richness than T1, T3 and T8 (p-value < 0.05) and T2 (p<0.1); T5 presented higher allelic richness than T1 (p<0.05) and T3 (p<0.1); T6 presented higher allelic richness than T1 (p<0.1); and finally T2 presented higher allelic richness than T1 (p<0.05). Considering allelic richness as an indicator of diversification [41], one may hypothesize that sublineages having significantly higher allelic richness are older; an observation which is particularly clear for T4 and T7 which appear as being older than T1 to T3. This assumption will be assessed in our future investigations based on WGS data of respective sublineages.

## Conclusions

This study explored for the first time 24-loci MIRU-VNTR based population structure of the so-called T group *M*. *tuberculosis* isolates from several countries around the world, and fetched new evidence about their phylogenetic structure into eight putative sublineages by phylogenetic and Bayesian analyses. Our results showed that 5 out of 8 sublineages seemed phylogeographically well-defined. This genetic structure now needs to be further validated by applying other genotyping approaches cumulating robustness of different methodss. We plan to start with an initial screening on a worldwide dataset using identical VNTR markers, followed by high throughput approaches avoiding homoplasy events like SNPs calling based on WGS, or Core Genome MLST (cgMLST). These studies should allow relevant worldwide exploration of evolutionary history of sublineages studied herein.

## Supporting information

S1 Fig MST illustrating evolutionary reliationship between *M*. *tuberculosis* T sublineages using spoligotypes markers. (A) MST according to sublineages as defined in the SITVIT2 database, (B) MST according to geographical areas. The complexity of the lines denotes the number of spacer changes between two patterns; the size of the circle is proportional to the total number of isolates sharing same pattern.
(TIF)

S2 Fig **Selection of appropriate K value by calculation of (A) ln P(D|K) and (B) delta K (Evanno method) for STRUCTURE analysis, and (C) DIC for TESS analysis.** Congruent value is observed at K = 8 for both approaches.
(TIF)

S3 Fig TESS Ancestry coefficient (Q-matrix) of *M*. *tuberculosis* T sublineages displayed spatially by universal kriging. Q-matrix are represented on (A) a single map, or (B) separate maps for each K, density of colors increasing with ancestry coefficient; black dots represent spatial coordinates of individuals.
(TIF)

S4 Fig MST based on 24-loci MIRU-VNTR illustrating evolutionary relationships of the *M*. *tuberculosis* T sublineage isolates (n = 607) prelabeled as T1 to T8 based on previous TESS analysis. Strains in intermediate position between sublineages are indicated as Int. The complexity of the lines denotes the number of allele/spacer changes between two patterns while the size of the circle is proportional to the total number of isolates sharing same pattern.
(TIF)

S5 Fig UPGMA based on 24-loci MIRU-VNTR of the *M*. *tuberculosis* T isolates (n = 607) compared to MIRU-VNTRplus database. A) Analyses with T1 to T4 isolates and B) analyses with T5 to T8 isolates.
(POT)

S1 Table Global dataset used in this study for the 607 *M*. *tuberculosis* T isolates. Countries names are defined by ISO 3166–1 alpha-3 code.
(XLS)

S2 Table Dunn’s test results. (XLSX)

S3 Table Percentage of allele copy number of 24-loci MIRU-VNTR markers in T1 to T8 *M*. *tuberculosis* isolates. ND: not done. (XLSX)
