# Supplementary figures and images for: Bayesian population structure analysis reveals presence of phylogeographically specific sublineages within previously ill-defined T group of Mycobacterium tuberculosis
Source: PLoS One. 2017 Feb 6;12(2):e0171584. doi: 10.1371/journal.pone.0171584 (PMC5293260; doi:10.1371/journal.pone.0171584)

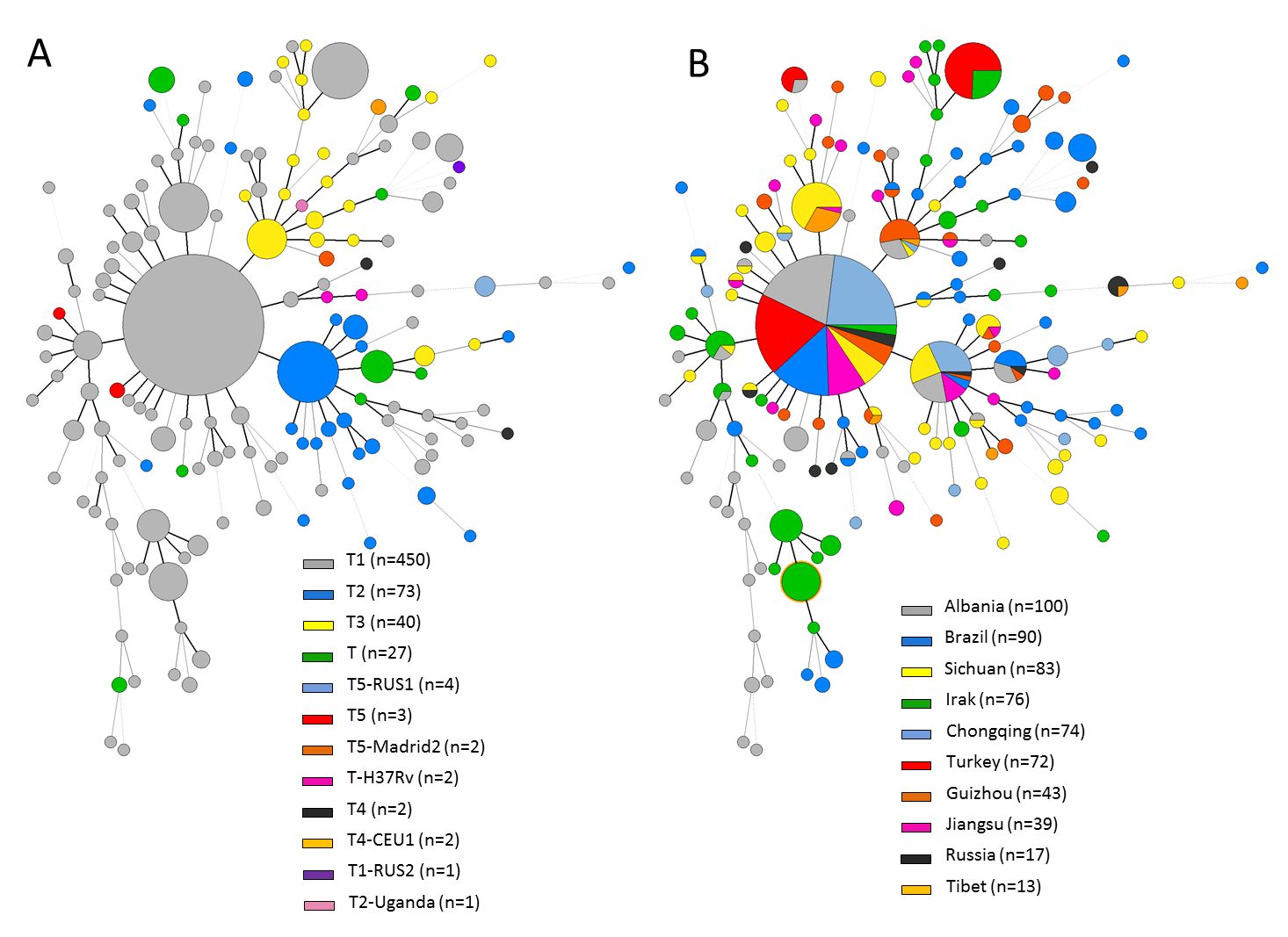

Supplement: S1 Fig — (A) MST according to sublineages as defined in the SITVIT2 database, (B) MST according to geographical areas. The complexity of the lines denotes the number of spacer changes between two patterns; the size of the circle is proportional to the total number of isolates sharing same pattern. (TIF) [file pone.0171584.s001.tif]

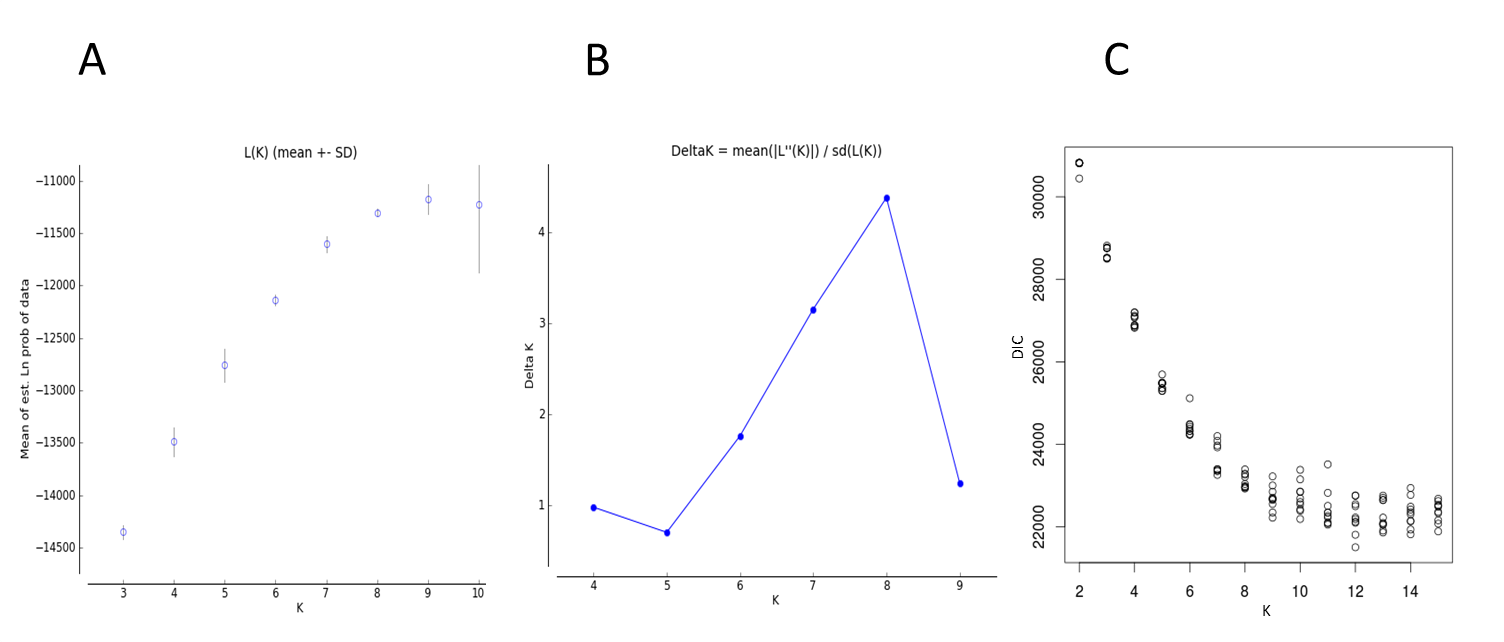

Supplement: S2 Fig — Selection of appropriate K value by calculation of (A) ln P(D|K) and (B) delta K (Evanno method) for STRUCTURE analysis, and (C) DIC for TESS analysis. Congruent value is observed at K = 8 for both approaches. (TIF) [file pone.0171584.s002.tif]

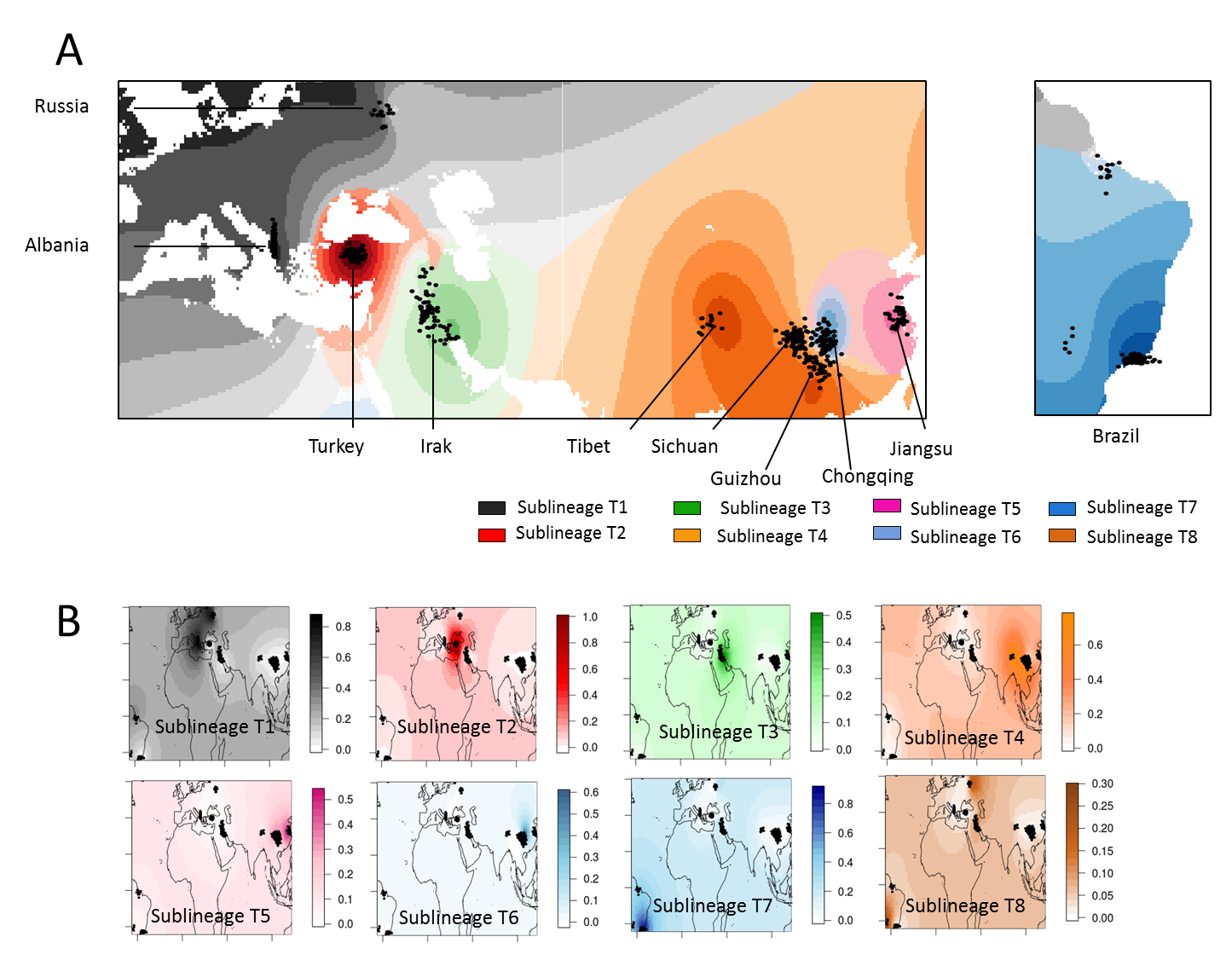

Supplement: S3 Fig — Q-matrix are represented on (A) a single map, or (B) separate maps for each K, density of colors increasing with ancestry coefficient; black dots represent spatial coordinates of individuals. (TIF) [file pone.0171584.s003.tif]

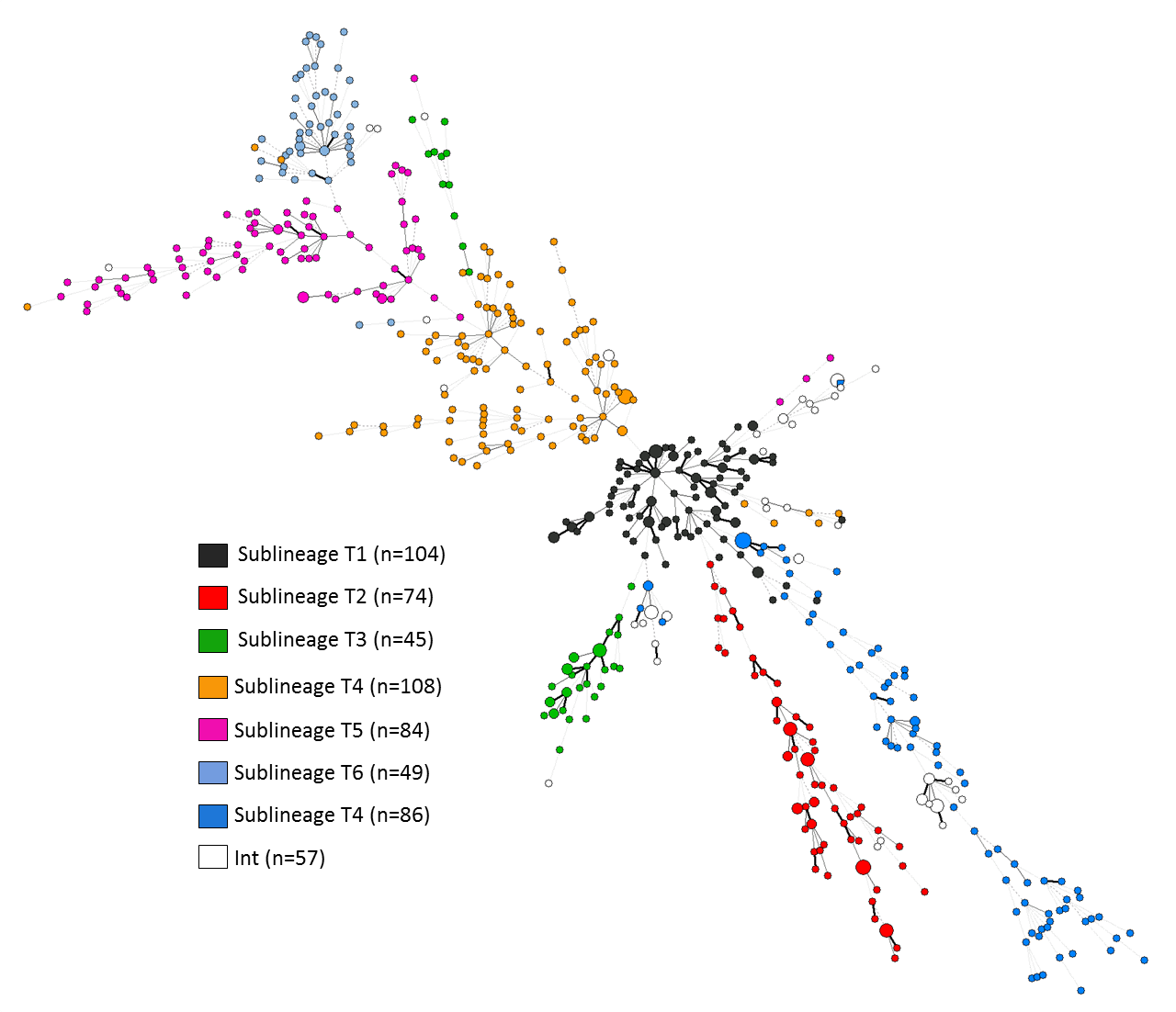

Supplement: S4 Fig — Strains in intermediate position between sublineages are indicated as Int. The complexity of the lines denotes the number of allele/spacer changes between two patterns while the size of the circle is proportional to the total number of isolates sharing same pattern. (TIF) [file pone.0171584.s004.tif]

## Slide 1
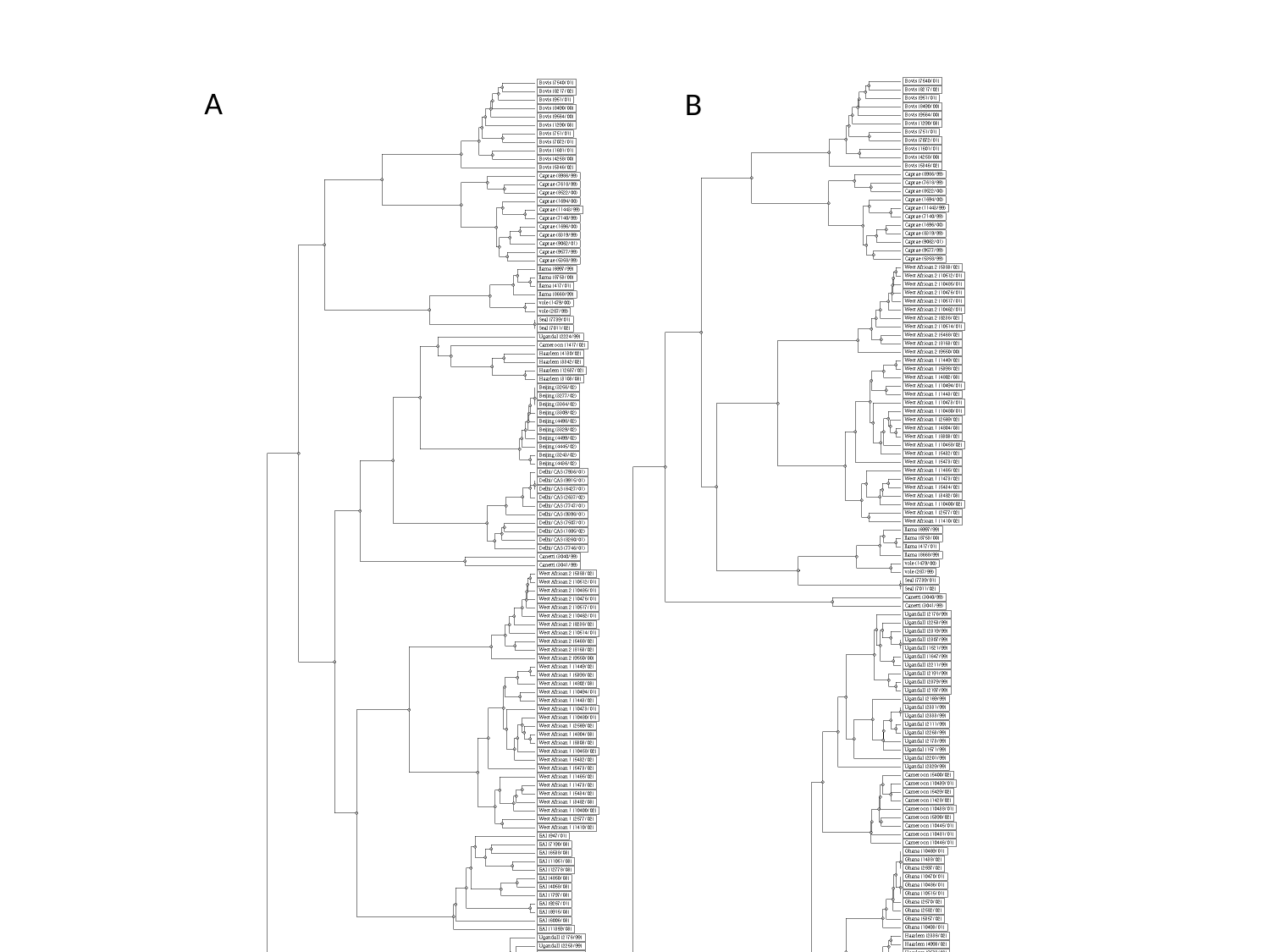

A
B

Supplement: S5 Fig — A) Analyses with T1 to T4 isolates and B) analyses with T5 to T8 isolates. (POT) [file pone.0171584.s005.pot]
